# Empirical Evidence of Environmental Technologies, Renewable Energy and Tourism to Minimize the Environmental Damages: Implication of Advanced Panel Analysis

**DOI:** 10.3390/ijerph20065118

**Published:** 2023-03-14

**Authors:** Ghazala Aziz, Suleman Sarwar

**Affiliations:** 1 Department of Business Administration, College of Administrative and Financial Sciences, Saudi Electronic University, Jeddah 93499, Saudi Arabia; g.aziz@seu.edu.sa; 2 Department of Finance and Economics, College of Business, University of Jeddah, Jeddah 23218, Saudi Arabia

**Keywords:** extended-STIRPAT, MENA, Saudi Vision 2030, COP26

## Abstract

The motivation behind this research is to investigate the determinants of the ecological footprint in MENA countries and find appropriate solutions. We updated the STIRPAT model and applied sophisticated panel techniques to data from 1996 to 2020. According to the findings, economic expansion along with urbanization and tourism is to blame for these countries’ huge environmental footprints. In addition, when it comes to environmental degradation remedies, environmental innovation and the use of renewable energy play an important role in minimizing these environmental externalities. The results of post Saudi Vison 2030 analysis confirmed the significance of urban population and renewable energy in minimizing the environmental footprint. In light of the findings, it is advised that policymakers should revise the legislative framework to attract not only private sector investment, but also foreign investment to utilize the full potential of renewable energy generation.

## 1. Introduction

Over the last few decades, rapid economic growth and the massive energy consumption required to support this growth, through electricity generation, industrialization, urbanization and transportation, has created environmental challenges to the world [1,2,3], and global CO_2_ is predicted to rise by 30% in 2030 compare with 2010 levels [4]. This alarming situation has urged all nations to reduce their energy usage, with decarbonization as the primary aim. The participants of COP26 at the Glasgow Climate Conference have joined hands to minimize the environmental pollution to help reduce the global temperature by 1.5–2 °C [5]. The shift in the global climate has caused the catastrophic ecological difficulties that raises the risk of sustainable development. Recently, a number of studies have been caried out to find the significant causes and solutions for the environmental gasses. Despite this research, there is no consensus developed; however, this study is a significant attempt to offer an environmental solution.

**Figure 1:**
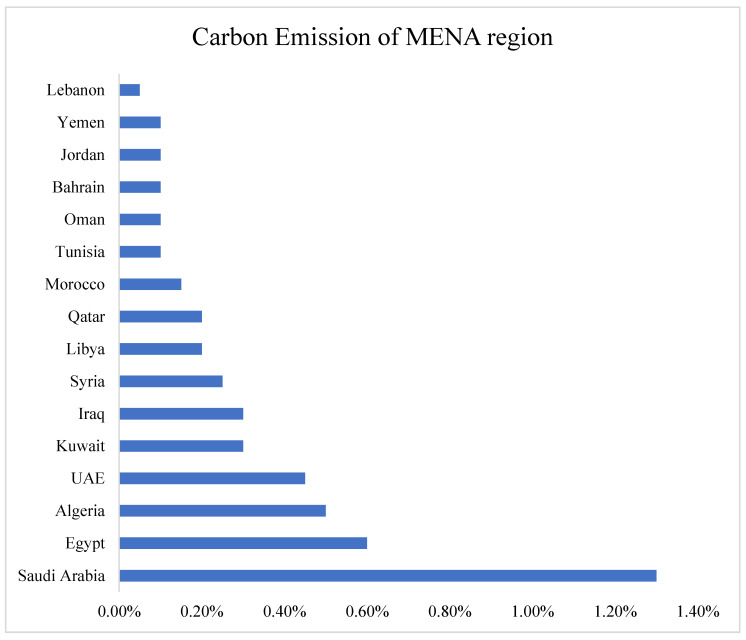
Carbon emission in the MENA region [16].

The Middle East and North Africa (MENA) nations are susceptible to the effects of climate change because of their inherent vulnerability to severe climatic conditions, exceptionally elevated temperatures, scarcity of water resources, etc. Due to the scarcity of water, most of the MENA countries, especially the gulf countries, have limited green areas, which further exacerbates the ecological challenges in this region [6]. These conditions are responsible for further climate challenges, such as significant changes in the annual and seasonal temperature. Consequently, in the recent era, the MENA region has witnessed a substantial rise in temperature as compared to the global temperature [7]. The main reason behind all these issues is the rising level of carbon emissions in the region. Figure 1 shows that carbon emission in the MENA region has been increasing continuously for many decades, and this situation needs immediate attention.

Researchers recognize the severity of the ecological footprint issue and are trying to explore options to solve it. While investigating the factors behind ecological footprint and possible solutions, Ref. [8] proposed a workable solution regarding domestic and foreign innovations. Another possible solution put forward from researchers in different countries is renewable energy [9,10,11]. According to them, carbon emissions from fossil fuels are the major contributor to the environmental footprint, and renewable energy is a potential remedy. In view of [12,13], human development can help reduce the ecological footprint. However, Refs. [14,15] argued that financial development is much more critical for countries that are trying to reduce their ecological footprint. All these studies use reliable data and proper methodology to find a solution for ecological issues.

Nonetheless, the problem is not only prevalent, but also growing significantly. As a result, more research into the aspects that contribute to ecological footprints and potential remedies is needed, and the current study is an attempt to add some critical factors to this conversation. More is research is needed; there is a void in the literature regarding the study of the MENA region. There has been no research in the region; hence, the current study fills the vacuum with empirical data from MENA countries. 

The above discussion indicates some vital contributions from this study. Firstly, we use data from the MENA region to explore the factors behind the high ecological footprint in these countries. Their ecological footprints have been steadily expanding, mainly because practically all of them require natural resources to build their economies. In 2016, the worldwide average ecological footprint per capita was 2.75 global hectares, of which 60% or 1.65 hectares was connected to carbon footprints, whereas removing carbon emissions required 4.15 hectares of land per person—roughly twice the global average [17]. To summarize, nations in the MENA region face not just carbon dioxide emissions, but also environmental challenges such as deforestation, overfishing, and water scarcity. Hence, it is necessary to find solutions for these countries to combat environmental challenges, including ecological footprint. 

The second contribution is the introduction of renewable energy consumption as a solution for the problem of ecological footprint in the MENA region. MENA has abundant solar resources that can produce green hydrogen from renewable power. According to the World Bank, the region has the most excellent photovoltaic power potential in the world, with the ability to create more than 5.8 kWh per square meter daily. By 2050, 83 GW of wind and 334 GW of solar power are expected to be installed, boosting wind and solar participation from 1% and 2%, respectively, to 9% and 24%. Renewable energy sources have many environmental advantages over hydrocarbons [18]. Most renewables are ecologically beneficial, since their life-cycle CO_2_ emissions are far lower than those of fossil fuels. Renewable energy sources such as solar, wind, and hydro produce no emissions, whilst other energy sources such as geothermal and biomass produce much lower emissions than traditional hydrocarbon-based fuels. Using and deploying various renewable energy sources in the MENA region can significantly alter the region’s economic and environmental dynamics. It is also essential, given the environmental and economic consequences of the region’s consistently increasing oil and gas consumption [19].

Thirdly, the study contributes to the existing literature through an emphasis on the significance of Saudi Vision 2030. The Saudi Vision 2030 is well received in the domain of economy and environment, as well as other green initiatives. The announcement of Saudi Vison 2030 has motivated the other regional countries to offer similar plans to counter their economic and environmental challenges. However, we use Saudi Vison 2030 as a base policy to investigate the changes in the region to curtail the environmental footprint.

The points mentioned above result in the creation of two primary objectives for this research. The first is to empirically investigate the determinants and solutions for an ecological footprint in the MENA region. In doing so, the role of the latest STRIPAT model in the region will be studied. Moreover, the significance of the Extended STRIPAT model will be checked by using robust analysis. The second is to investigate the significance of Saudi Vision 2030 in reducing the environmental challenges in the MENA region.

## 2. Literature Review

### 2.1. Urbanization and Ecological Footprint

As the world modernizes, the trend towards urbanization increases. However, due to increased urbanization, threats to the environment get severe, especially concerning ecological footprint [20]. The hypothesis of urbanization’s negative impact on the ecological footprint in African countries was tested by utilizing data from 2000 to 2007. Their results showed increased pressure on biodiversity because urbanization in the selected countries was increased. This inverse relationship between urbanization and ecological footprint is valid in African countries and Bangladesh. A study done by [21] collected data from 1990 to 2016 in Bangladesh and proved though different analysis techniques that the country’s ecological footprint had increased. They argue that urbanization is the main reason behind this rapid increase in ecological footprint. Likewise, Ref. [22] investigated the same nexus in Gulf Cooperation Council (GCC) countries. By investigating data from 1995 to 2017, they also came up with the same conclusion, that ecological footprint is the outcome of urbanization. The Ref. [23] added to this discussion by taking data from the Sindh and Baluchistan provinces from 2016 to 2017. They said that due to increased urbanization, the demand for resources is highly increased, leading to a high level of consumption. This puts pressure on ecological resources and makes urbanization a significant problem that causes a high ecological footprint.

### 2.2. Economic Growth and Ecological Footprint

Countries worldwide try to increase economic growth but neglect the adverse outcomes of high economic growth. Hence, researchers shed light on this issue by conducting empirical studies. In this regard, Ref. [10] used massive data from 166 nations and divided the sample into three parts based on income level. Their analysis suggests that although all countries face the issue of ecological footprint, low-income countries are under less pressure to show improvement in economic growth. Also, Ref. [24] tried to check whether economic growth in oil-producing countries is also creating ecological issues. They used data from oil-producing nations from 1999 to 2017 and applied the dynamic panel data technique. Their results show that the ecological footprint in these countries is due to a high level of economic growth.

Additionally, an extensive data set from 120 countries was utilized by Ref. [11] to find the causes and solutions for ecological problems. They found that economic growth is the main problem in the selected countries, in addition to other factors, because it increases the ecological footprint. The analysis of Ref. [25] is worth mentioning in the discussion regarding ecological footprint because they used data from Japan, a highly developed country, to find if developed nations also have the problem of ecological footprint. They used data from 1965 to 2017 and applied the Quantile ARDL model. They found that economic growth and ecological footprint are significantly and positively related in low and high quantiles. Also, this positive impact of economic growth is valid in the short and long run.

### 2.3. Renewable Energy and Ecological Footprint

The debate regarding the problem of ecological footprint is not complete without mentioning renewable energy consumption as a viable solution. Empirical evidence also shows the benefits of renewable energy for preserving ecological resources. Ref. [26] conducted a study on BRICS-T countries by analyzing data from 1990 to 2018 to explore the benefits of renewable energy consumption. Their results suggest that the ecological footprint can be reduced significantly through the consumption of renewable energy. Another study by Ref. [27] used data from South Asian economies from 1990 to 2017. Through the latest econometric techniques, they also found that the solution to the problem of ecological footprint lies in the consumption of renewable energy because the least amount of carbon is emitted through this type of energy. The benefits of renewable energy consumption are also valid for N-11 countries; Ref. [9] used empirical data from these countries from 1990 to 2018 to investigate the significance of renewable energy consumption. Through the quantile regression technique, they found that the problem of ecological footprint in these countries can be controlled by consuming renewable energy. Along the same line, Ref. [25] also suggests that renewable energy consumption is the best option for Japan to control the problem of its ecological footprint.

### 2.4. Tourism and Ecological Footprint

Tourism plays a vital role in the economic development of economies. However, there are some problems related to tourism in terms of the environment. To investigate this, Ref. [28] conducted research in Pakistan using data from 1980 to 2017. They applied the ARDL and Bayer and Hanck tests and found that tourism is connected to ecological footprints through an inverted U-shaped curve. Additionally, Ref. [29] utilized data from 13 Mediterranean-protected destinations to check if the ecological footprint in these areas is the outcome of tourism. They suggest that in selected areas, tourism leads to an ecological footprint. A detailed study was done by [30], who divided the data according to countries’ income levels and made four groups of countries. Using data from 1995 to 2019, they found that tourism activities reduced resource depletion. Hence, tourism should be promoted to reduce its ecological footprint. Also, Ref. [31] used a massive data set from 123 countries to check the determinants of an ecological footprint. Their analysis proved that an increase in tourism adds to the depletion of natural resources, enhancing the overall ecological footprint.

## 3. Data and Methodology

### 3.1. Data

This study focused on MENA counties, including “Algeria, Bahrain, Egypt, Iran, Iraq, Israel, Jordan, Kuwait, Lebanon, Libya, Morocco, Oman, Qatar, Saudi Arabia, Syria, Tunisia, United Arab Emirates and Yemen”. Data from 1996 to 2020 was used in the analysis by setting ecological footprint as the dependent variable. The study’s independent variables were economic growth, tourism, environmental innovation, urban population, and renewable energy consumption. While investigating environmental technology and renewable energy, it is important to distinguish these terms and provide a multicollinearity analysis. Environmental technology commonly deals with the innovation of carbon capture, carbon storage, energy storage, transport network, biomass, recycling facilities, waste of fuel, etc., in contrast to renewable energy from wind, solar, geothermal, hydro, energy storage etc. However, it is important to cover both green variables to examine the influence of each variable to minimize the environmental externalities. Moreover, we reported the multicollinearity analysis in Table 1, Section B, which clearly indicates the non-existence of multicollinearity between the variables. The description of variables and sources is presented in Table 1, Section A. 

### 3.2. Methodology

Ref. [32] introduced the IPAT model in the 1970s, and it has become the most adopted model since then. The main reason for the high acceptance of the model is that it can understand environmental changes based on human factors. The three main drivers in this model are “population, urbanization, and technology”, and Equation (1) represents the model.

(1) I=P×A×T

where I=EF,P=UP,A=EG,T=ET

Later, this model was revised [32], and the resultant model is called STRIPAT, as shown in Equation (2). The reason for revising the model is the previous model’s inability to test the hypothesis reliably. Also, it took effort to differentiate factors based on their relative importance. Alongside this, the new model incorporates the “Environment Kuznets Hypothesis”, making it more reliable.

(2) $$I_{it}=\varphi_{0}P_{it}^{\rho_{1}}A_{it}^{\rho_{2}}T_{it}^{\rho_{3}}\mu_{it}$$

Equation (2) is ecological footprint, P is population, A is economic growth, T is technology, t is a time dimension, ρ is a coefficient parameter, μ is white noise, and i is country. In this study, the proxy for P is the urban population, and for T, it is environmental technology. The relationship between variables is presented in Equations (3)–(6). When these equations are transformed into the STRIPAT model, Equations (7)–(14) are formed.

Model-1 focuses on STRIPAT:

(3) EF=f (UP, EG, ET)

Model-2 focuses on STRIPAT, including *RE*:

(4) EF=f (UP, EG, ET, RE)

Model-3 focuses on STRIPAT including *RE* and *TOURISM*:

(5) EF=f (UP, EG, ET, RE, TOURISM)

where, EF is ecological footprint, UP represents urban population (urbanization), EG mentions gross domestic product, ET shows environment-related technology, RE is renewable energy consumption, and TOURISM represents tourism.

(6) $$EF_{it}=\varphi_{0}UP_{it}^{\rho_{1}}EG_{it}^{\rho_{2}}ET_{it}^{\rho_{3}}\mu_{it}$$

(7) $$lnEF_{it}=\rho_{0}+\rho_{1}UP_{it}+\rho_{2}EG_{it}+\rho_{3}ET_{it}+\varepsilon_{it}$$

(8) $$EF_{it}=\varphi_{0}UP_{it}^{\rho_{1}}EG_{it}^{\rho_{2}}ET_{it}^{\rho_{3}}RE_{it}^{\rho_{4}}\mu_{it}$$

(9) $$lnEF_{it}=\rho_{0}+\rho_{1}UP_{it}+\rho_{2}EG_{it}+\rho_{3}ET_{it}+\rho_{4}RE_{it}+\varepsilon_{it}$$

(10) $$EF_{it}=\varphi_{0}UP_{it}^{\rho_{1}}EG_{it}^{\rho_{2}}ET_{it}^{\rho_{3}}RE_{it}^{\rho_{4}}TOURISM_{it}^{\rho_{5}}\mu_{it}$$

(11) $$lnEF_{it}=\rho_{0}+\rho_{1}UP_{it}+\rho_{2}EG_{it}+\rho_{3}ET_{it}+\rho_{4}RE_{it}+\rho_{5}TOURISM_{it}+\varepsilon_{it}$$

In order to reduce the skewness of the distribution and make equations tractable, the log form of “STRIPAT” is used in this study [33]. Ecological footprint in these countries is the dependent variable, whereas independent variables include urban population, economic growth, environmental technology, renewable energy consumption, and tourism. Table 1 presents the details regarding the data description and sources.

## 4. Econometric Approaches

First, we have to provide the basic statistics of the variables to understand the descriptives, which helps to explore the uneven occurrence in the data series, such as outliers. Afterward, we have to examine the homogeneity in the data, which is useful for providing the dynamics of the data structure. Secondly, as we employ the panel data, we have to examine the cross-sectional dependence. In case of no existence of cross-sectional dependence, we go for the first-generation unit root and cointegration tests. In contrast, the existence of cross-sectional dependence will lead us towards the applicability of second-generation unit root test. 

We used the Pesaran CD and Pesaran scaled-LM tests to examine the presence of second cross-sectional dependence. For the second-generation unit root test, we had to apply the second-generation Cross-Sectionally Augmented IPS (CIPS) and Cross-Sectionally Augmented Dicky-Fuller (CADF) tests. While confirming the stationarity at first difference, we applied the cointegration test, which helps to find the existence of long run relationship between independent and dependent variables. For this purpose, we used the second-generation cointegration test and Westerlund cointegrations, which are useful for confirming the existence of long run relationship between the studies variables. 

In the presence of a long-run relationship, the study applied the relevant regression techniques to explore the impact of independent variables on the dependent variable. However, we used multiple regression techniques to reaffirm the empirical results of this study, such as (i) quantile via moment, (ii) Driscol Karaay regression, (iii) estimations with threshold, etc. 

### 4.1. Quantile Regression

Quantile regression was introduced by Ref. [34], in order to examine distributional and heterogeneous influences across quantiles. Initially, Ref. [35] presented this regression approach to estimate the conditional median or a number of alternative quantiles of the output responses when particular values of the exogenous factors are present, as opposed to ordinary least-squares regressions, which provide estimates of the conditional mean of the endogenous variable when specific values of the exogenous variables are present. Quantile regressions are considerably more tolerant of outliers during estimation. In addition, it is especially applicable when the conditional means of two variables have little to no correlation.

In contrast, the “Method of Moments Quantile Regression” (MMQR) was employed in this work to address the issue of unobserved heterogeneity. By detecting the conditional heterogeneous covariance effects of ecological footprint determinants, the MMQR methodology allows individual influences to influence the whole distribution rather than just the averages. The following equation was utilized for the estimation of conditional quantiles $Q_{Y}\left(\tau |X\right)$:

(12) $$Z_{it}=\beta_{i}+{Y^{\prime }}_{it}\gamma +\left(\theta_{i}+{X^{\prime }}_{it}\epsilon \right)U_{it}$$

where $P\left\{\theta_{i}+{X^{\prime }}_{it}\epsilon >0\right\}\;$= 1. $(\beta ,\gamma^{\prime },\theta ,\epsilon^{\prime }{)}^{\prime }$ are parameters, X is a k-vector of Y, a differentiable transformation with l element given by

(13) $$X_{l}=X\left(Y\right),\;l=1,\ldots k$$

where $Y_{it}$ is identically distributed independently of i and t. $U_{it}$ is independent, identically distributed across individuals (i) and time (t), and orthogonal to $Y_{it}$. Hence, the below equation is the extended form of Equation (13).

(14) $$Q_{Y}\left(\tau |Y_{it}\right)=\left(\beta_{i}+\theta_{i}q\left(\tau \right)\right)+{Y^{\prime }}_{it}\gamma +{X^{\prime }}_{it}\epsilon q\left(\tau \right)$$

In Equation (14), ${Y^{\prime }}_{it}$ is the independent variable’s vector, $Q_{Y}\left(\tau |Y_{it}\right)$ is the dependent variable’s quantile distribution, and $\beta_{i}\left(\tau \right)=\beta_{i}+\theta_{i}q\left(\tau \right)$ is a scalar coefficient. 

### 4.2. Threshold Regression

We used the threshold regression technique to examine the non-linearity of the relationship between urbanization and the ecological footprint. Additionally, it was examined using the “unconditional fixed-effects quantile estimation approach” to see whether urbanization varied at various ecological footprint levels. In doing so, the following equation uses the fixed-effect panel threshold model:

(15) $$Z_{it}=\beta_{i}+\delta q_{it}+\theta_{i}Y_{it}+\epsilon_{it}$$

where $Z_{it}$ = ecological footprint, i = country, t = time in which (I≤i≤N) and (I≤t≤N). $\beta_{i}$ and $\epsilon_{it}$ are the fixed and random effect errors, whereas $q_{it}$ is the urbanization in country i and time t. Also, the k-dimensional vector of control variables is $X_{it}$. To operationalize Equation (18), Equation (19) is built on one threshold.

(16) $$Z_{it}=\beta_{i}+\delta_{1}q_{it}I\left(q_{it}\leq \gamma \right)+\delta_{2}q_{it}I\left(q_{it}>\gamma \right)+\theta_{i}Y_{it}+\epsilon_{it}$$

where γ = threshold, *δ*_1_ and *δ*_2_ = slopes of threshold variables $\delta_{1}$ and $\delta_{2}$. Equation (17) is used to check the soundness of the model.

(17) $$F_{1}=\frac{S_{0}-S_{1}}{\hat{\varphi^{2}}}$$

where $S_{0}$ displays the residual sum of squared errors of the linear model, $S_{0}$ and $S_{1}$ display the residual sum of squared errors of the threshold model, $S_{1}$, respectively, and the residual variance of the threshold model is shown by $\hat{\varphi^{2}}$. However, bootstrapping of estimates is required to get the asymptotically correct p values. The following equation illustrates the null and alternative hypotheses in our study:

(18) $$H_{0}=\delta_{1}=\delta_{2};\;H_{1}=\delta_{1}\neq \delta_{2}$$

### 4.3. Panel Granger Causality Test

The below-mentioned equation is used to check the granger causality between variables:

(19) LNEF=∫(LNUP, LNGG, LNET, LNRE,LNTourism)

As mentioned above, the variables’ stationarity is the primary condition of the paradigm. Granger Causality was initially described by [36,37] and utilized in several studies. Nonetheless, Ref. [38] introduced the “Half Panel Jackknife (HPJ)” method to address the difficulties of parameter bias and size distortion. In this work, the Granger approach identified and utilized by [39] is implemented utilizing the following equation:

(20) $$y_{it}=\beta_{0i}+\sum_{o}^{p}=1\beta_{pi}y_{it-p}+\sum_{q}^{Q}=1\delta_{qi}X_{it-q}+\epsilon_{it}$$

where N is the number of nations and ranges from 1 to N, T is the time, and t ranges from 1 to T. In addition, $\epsilon_{it}\sim N\left(0,\sigma^{2}\right)$, and $\beta_{pi}$ are the coefficients of the autoregressive term, whereas $\delta_{qi}$ is the coefficient of the feedback term. To convert the above equation to vector form, use the following setting:

(21) $$\begin{array}{c} (1,y_{it-1},\ldots ,y_{it-p}{)}^{\prime }\mathrm{as}\;z_{it},\;(1,X_{it-1},\ldots ,X_{it-q}{)}^{\prime }\mathrm{as}\;x_{it},\;\beta_{i}=(\beta_{0i},\ldots ,\beta_{pi}{)}^{\prime },\;\delta_{i}=(\delta_{0i},\ldots ,\delta_{pi}{)}^{\prime },\;y_{i}=(y_{i1},\ldots ,y_{iT}{)}^{\prime },\;\\ \epsilon_{t}=(\epsilon_{1i},\ldots ,\epsilon_{Ti}{)}^{\prime }. \end{array}$$

The below equation is formed now:

(22) $$y_{i}=Z_{i}\beta_{i}+X_{i}\delta_{i}+\epsilon_{i}$$

Homogeneity of $\delta_{i}$ is assumed, making the above equation as follows:

(23) $$y_{i}=Z_{i}\beta_{i}+X_{i}\delta +\epsilon_{i}$$

The issue of parameter bias is solved by [39] by calculating the value of δ as follows:

(24) δ˜=2δ^−12(δ^12+δ^21)=δ^+((δ^−12(δ^12+δ^21))

The hull hypothesis argues that there is no Granger causation between variables, whereas the alternative hypothesis is that Granger variables cause each other. There are numerous advantages of this approach; some significant advantages include overcoming the “Nickell” bias and compensation for coefficient bias when T < N. Hence, this technique efficiently examines the connection between variables in this study’s homogenous and heterogeneous panels. Second, many challenges that diverse nations encounter require a global strategy. More particularly, this strategy allows for cross-sectional dependence, which enhances the validity of panel data inference. Last but not least, panel data models increase the quality and volume of data, enabling a more accurate model inference to take the impacts of omitted variables into account [40]. In contrast to the benefits of Granger causality, there are limitations, such as: (i) Granger causality is unable to measure the relationship between the variables, (ii) it is not able to be performed on non-stationary data series, (iii) in case of interdependence between two or more variables, it is unable to provide the forecasted results.

## 5. Results

### 5.1. Preliminary Test

#### 5.1.1. Descriptive Statistics

Descriptive statistics are presented in Table 2, where the highest mean is for environmental technology, and the lowest mean is for ecological footprint. It can be seen that the standard deviation is highest for renewable energy and lowest for economic growth. In terms of skewness, all variables are positively skewed. 

#### 5.1.2. Diagnostic of Variables

Before proceeding with the study, it is necessary to check the cross-sectional dependency, for which the Pesaran’s CD and Pesaran scaled-LM tests were applied, and the findings are shown in Table 3. The values for all variables under the two tests are significant, which means that different shocks are transferred to other countries and are not limited to a single country.

#### 5.1.3. Test for Slop Homogeneity

In this study, the homogeneity test of [41] was employed in addition to the tests of [42]. The results are presented in Table 4. All values in all models are significant at 1% significance. This confirms the heterogeneity of the panel data and coefficients and the difference of slops across the country. It can be said that other countries do not have the same pattern in structure of socioeconomic environment in each country. Hence, the estimations will be unbiased without homogeneity restrictions [43]. 

#### 5.1.4. Unit Root Tests

To confirm that the data is stationary, the unit root test is essential in the current study; “Cross-Sectionally Augmented IPS (CIPS) and Cross-Sectionally Augmented Dicky-Fuller (CADF)” are used for this purpose. Table 5 reports the test results where all variables have significant coefficients at 1st difference. The order of the variable’s integration is I (1), which leads to the decision to use the Westerlund co-integration test. 

#### 5.1.5. Westerlund Co-Integration Test

In this study, the Westerlund co-integration test is utilized to check the co-integration’s existence, and Table 6 reports the results. It is evident that co-integration is present among the variables because all *p*-values are significant, and the null hypothesis is rejected. 

### 5.2. Regression

#### 5.2.1. Quantile via Moment 

The relationship between independent and dependent variables is investigated through Method of Moment quantile regression [34], and Table 7 shows the results. In Model 1, the main focus is on ecological footprint through three main factors. In the case of the urban population, coefficients are significant in all quantiles. However, it is essential to note that the impact on ecological footprint is negative in lower quantiles and positive in upper quantiles. The impact of urban population is highest in the eighth quantile, where a 1% increase in urban population results in an increase of 4.66% in ecological footprint. Thus, economic expansion has a favorable impact on the ecological footprint in all quantiles. Nevertheless, the magnitude of impact increases as we move to higher quantiles. In MENA countries, economic growth is the main factor behind the high ecological footprint, ass it is evident that in the 0.7, 0.8 and 0.9 quantiles, the coefficients are 9.344, 7.554 and 9.854, respectively, whereas, in the lower and middle quantiles the coefficients vary from 0.326 to 5.891. In contrast to this, we find mix evidence regarding magnitude, but the level of significance is still higher in the upper quantiles at a level of 1 percent. Ref. [30] also found a positive relationship between economic growth and ecological footprint. These results suggest that the MENA countries with low ecological footprints are less affected by economic growth. In the case of environmental technologies, the impact is significant and negative in most quantiles. Hence, in MENA countries, environmental technology is a viable solution to reduce the ecological footprint. Furthermore, Ref. [43] also argued that technical innovations can reduce the ecological footprint.

In Model 2, renewable energy was added to the equation. In this model, the urban population is significantly and positively related to the ecological footprint. The negative impact of urban population is only in the 10th and 20th quantile. This means that if the urban population increases by 1%, the ecological footprint increases by −5.103 to −3.098 percent. A study by [8] proved that urban population could enhance ecological footprint. In this model, environmental technology is also significantly and negatively related to ecological footprint. This impact is prominent from the 30th to the 90th quantile. However, the most substantial impact is in the 70% quantile, where a 7.6% reduction in ecological footprint is due to a 1% increase in environmental technology. As far as renewable energy consumption is concerned, it is significantly and negatively related to an ecological footprint in the 70th to 90th quantile from 4.001% to 7.001%. According to this result, MENA countries that have a high ecological footprint can benefit the most from renewable energy.

Model 3 incorporated tourism in the equation, where the urban population is significantly and negatively related to the ecological footprint in lower quantiles and significantly and positively related to the ecological footprint in upper quantiles. This means that urban population is not an issue for countries with low ecological footprints, but where the ecological footprint is high, urban population adds to the issue. In this model, economic growth is also significantly and positively related to the ecological footprint in all quantiles. For environmental technology, its relationship with the ecological footprint is significant and negative in only upper quantiles. Only countries with a high ecological footprint can benefit from environmental technology. Tourism is significantly and positively associated with an ecological footprint in four of the nine quantiles. This suggests that tourism is a significant factor behind the ecological footprint of MENA countries.

#### 5.2.2. Long-Run Analysis

**Figure 2:**
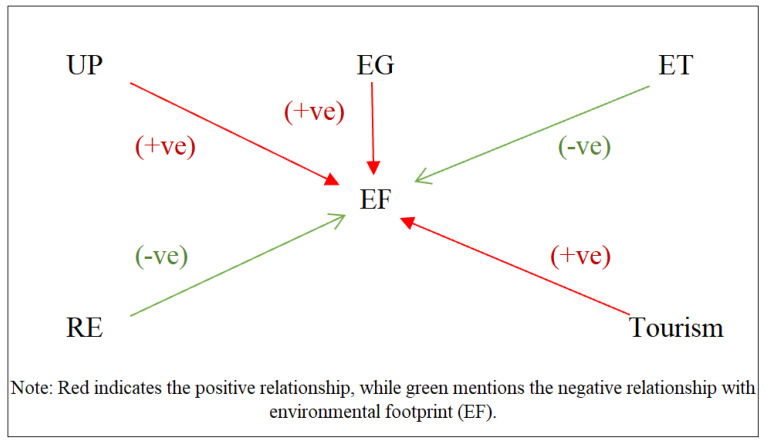
Graphical presentation of Driscol Karaay Regression results.

For comparison, Driscol Karaay regression was used, and Table 8 shows the results. The impact of urban population on the ecological footprint of MENA countries is significant and positive in all models, which means urban population enhances the ecological footprint. The highest coefficient was observed in model 3, where a 1% increase in urban population increases the ecological footprint by 1.693%. This result is aligned with the results of [8,44,45]. The following variable is economic growth, which is significantly and positively related to ecological footprint at a 1% significance level. Just like the urban population, economic growth has the highest impact on the ecological footprint in model 3, wherein a 2.304% increase in ecological footprint is due to a 1% increase in economic growth. However, environmental technology is significantly and negatively related to the ecological footprint in all models. The coefficient for environmental technology is largest in Model 2. It can be seen that a 1% increase in environmental technology decreases the ecological footprint by 0.709%. Refs. [46,47] discovered a link between environmental technology and environmental footprint. Surprisingly, renewable energy has no relationship with the environmental impact in any model. This indicates that MENA countries are not benefiting from renewable energy. This result is contrary to the findings of [9,48,49]. Also, in this regression, tourism is significantly and positively related to ecological footprint at a 5% significance level. The coefficient value is 0.418, meaning that a 1% increase in tourism increases the ecological footprint by 0.418%. Refs. [28,50] also found the same type of results in their analysis. The Driscol Karaay regression results are presented in Figure 2.

#### 5.2.3. Threshold Regression

In threshold regression, three thresholds are checked in case of rejection of the hypothesis regarding the single threshold. Table 9 presents the results of threshold regression, where at 11.251 of GDP, one threshold is evident at a 95% confidence interval between 10.071 and 10.109. Additionally, probability and F-statistics also confirmed the existence of one or more than one threshold between the association of GDP and ecological footprint. This proves the rejection of the linearity hypothesis. Following that, the second and third thresholds are estimated; however, probability and F-statistics show that only one threshold exists. As a result, a single threshold is employed, and the results are shown in Table 10.

According to Table 10, the coefficient for environmental technology is significant and negative at a 10% significance level in Model 1. A coefficient of −0.971 means a 1% enhancement in environmental technology reduces ecological footprint by 0.971%. Likewise, the coefficient for environmental technology in Model 2 is also negative and significant, suggesting that a 0.261% reduction in ecological footprint is due to a 1% increase in 0.261. In this model, renewable energy is significantly and negatively associated with an ecological footprint at a 10% significance level. A 1% increase in renewable energy consumption leads to a 0.082% reduction in ecological footprint. In Model 3, the coefficient for environmental technology is significant and negative at a 5% level of significance, whereas the coefficient for renewable energy is significant and negative at a 10% significance level. Hence, a 1% increase in environmental technology and renewable energy consumption reduces the ecological footprint by 0.262% and 0.015%, respectively. However, the coefficient of tourism is significant and positive at a 5% significance level with a coefficient value of 0.280. It can be said that a 0.280% increase in ecological footprint is a result of a 1% increase in tourism.

**Figure 3:**
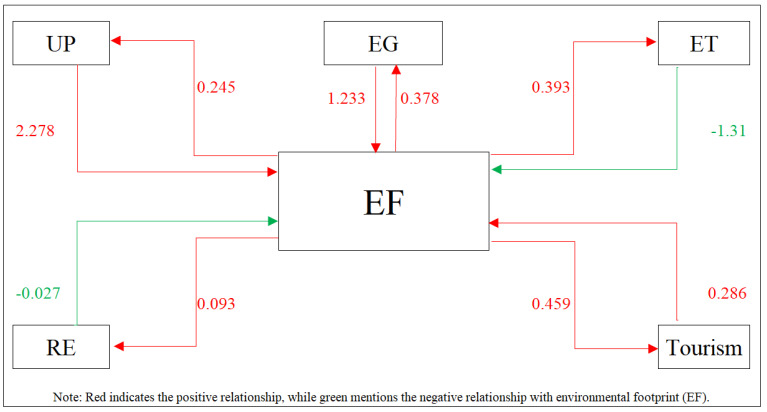
Graphical presentation of Juodis, Karavias and Sarafidis (2021) [39] Granger non-causality test.

Granger non-causality was checked in the final step of the analysis [51]. Results are presented in Table 11, where it is evident that the Granger-cause hypothesis is accepted for all considerations at 1% and 5% significance levels. The bidirectional causality is also confirmed between ecological footprint and the dependent variable and independent variables, including urban population, economic growth, environmental technology, renewable energy consumption, and tourism. As a result, because independent variables provide essential information, the ecological footprint can be estimated. The findings are presented in Figure 3.

### 5.3. Post Saudi Vision 2030

It is well evident that it is easy to obtain economic stability though regional economic stability rather than the growth of an individual country in a region [52]. However, it is important to discuss the impact of the economic and environmental agenda of a country on the regional countries. For this reason, we have focused on the well-publicized Saudi Vision 2030 that has gained tremendous appreciation around the world. In this section, we emphasized the post-Vison-2030 period and divide the dataset as in [53]. We used the data from 2016 and converted it into a monthly dataset to attain the minimum number of observations that are required by econometric estimations. Further, we employed the Markov chain Monte Carlo (MCMC) algorithm and converted it to a monthly dataset, as in [52,54]. Table 12 presents the regression results of post Saudi Vision 2030.

The findings of post Vison 2030 are quite surprising, as the urban population turned out to be significant and negative, but the magnitude was still very low. This indicates that urbanization did not further increase the environmental footprint. The reason can be seen in the enormous focus on environment by the governments, as was indicated in COP27 held in Egypt. Green campaigns by the authorities and at the public level have motivated the population to adopt green instructions to minimize environmental degradation. 

The coefficients of economic growth are still significant and positive, suggesting no fruitful findings through Vision 2030. Environmental technology had significant and positive coefficients, similar to previous results. However, the magnitude of environmental technology has increased, which highlights the fact that the increase in environmental technology has significantly minimized the environmental externalities. Surprisingly, renewable energy had a significant and negative relationship with environmental footprint, but the coefficients were very low. The reason for this change is due to the authorities’ focus towards the environment; however, there can be seen a modification in the energy source mix in these countries, especially in the post-Vision-2030 period, whereas, tourism is still a significant contributor to environmental externalities.

### 5.4. Discussion

Movement Method Quantile regression revealed a substantial negative relationship between environmental technology and ecological footprint in the upper quantiles or nations with high ecological footprints. However, a panel’s estimate of the environmental technology’s long-term impact verified that it minimizes the ecological footprint. Environmental technology that corresponds to the ecological principle and the law of ecological economics conserves resources and energy. It eliminates or reduces environmental effects and is crucial in determining an ecological footprint. It includes hydro, bioenergy, solar, wind, geothermal, marine, natural gas, and nuclear energy technologies. The development of green energy technology will significantly stimulate the clean and efficient use of fossil fuels, as well as the use of renewable energy, therefore reducing the environmental effect [47]. 

The impact of renewable energy consumption on the MENA region’s ecological footprint is negative in the upper quantiles. However, long-run estimates show that renewable energy did not impact the ecological footprint. The reason behind the insignificance of renewable energy concerning ecological footprint is explained in [55]. Renewable energy output in the MENA area will more than quadruple over the next five years, according to the International Energy Agency, as governments increase investment in green technologies and reduce reliance on hydrocarbons. According to the organization’s Renewables 2021 assessment, capacity in the area will exceed 32 GW by 2026, up from 15 GW presently. However, the MENA region lags well behind other regions that have adopted much greater measures to boost their renewable footprint and phase out fossil fuels [56]. Hence, more than just the consumption of renewable energy in this region is needed to show a significant impact on ecological footprint.

When it comes to the causes of environmental impact, economic expansion and urban population are at the top of the list. This is because the economy is excessively dependent on nonrenewable energy and natural resources. One of the significant reasons for a minor impact of renewable energy on environments is due to the dependence on nonrenewable energy for the energy mix. Most of these countries have nonrenewable natural resources that have been utilized to generate electricity for domestic and industrial consumption. These countries have already invested huge initial capital into production and distribution channels. However, in such case, these natural-resource-rich countries are reluctant to move towards fully renewable energy. Focusing on the countries with limited resources, the initial cost of installing renewable energy sources on a huge level is out of reach. Consequently, renewable energy is not playing role in countering the environmental challenges in these countries. 

Rapid economic expansion in the MENA nations has been associated with high economic growth rates, energy consumption, and CO_2_ emissions. Urban population is also related to high ecological footprint. People’s quality of life has improved due to expanded urbanization opportunities, higher earnings, and easier access to intercity transit due to owning a car. Urbanization promotes deforestation, polluted water, climate change, and population growth in urban areas, all of which have a considerable detrimental impact on the environment as a result of the increasing demand for durable products and the extensive use of electrical technology for quick services [21]. They also emphasized that, according to ecologists, an “ecological overshoot” occurs when a rise in population and urbanization disrupts the equilibrium between extraction and the regeneration of resources. The same is true for GCC nations, where expanding urbanization has led to a high demand for the exploitation of nonrenewable natural resources (oil and gas), resulting in a significant ecological impact.

In case of post Saudi Vision 2030 estimations, we saw the surprising effect of urbanization, wherein there were significant and negative coefficients. The findings confirmed that higher urbanization tends to reduce the environmental externalities in the MENA region. The reason may be the adoption of energy efficient, green and environmental technologies by the residents, which helps them to reduce energy consumption, which is effective in minimizing environmental damages. Moreover, the magnitude of environmental technologies and renewable energy consumption has boosted, which reflects the positive role of environmental technology and renewable energy to minimize the environmental externalities. However, tourism is still a main source of environmental pollutant gasses that need to be dealt with by policy makers.

## 6. Conclusions

The motivation behind this study was to investigate the determinants of the ecological footprints in MENA countries and also to find solutions for this problem. Through analysis, it was proven that the high ecological footprint in these countries is due to their economic growth, tourism activities, and increasing urban population. Environmental technologies and renewable energy usage proved to be the most effective solutions. The renewable energy potential of these nations is exceptional. However, they need to utilize it properly. According to [57], the total installed electricity capacity in MENA was roughly 185,000 MW at the end of 2012, with only about 19,000 MW (or 10%) coming from renewable sources. The output of renewable energy in the region is less than 4% of the total primary energy consumption, which contrasts sharply with the average global share of 17%. The MENA area is endowed with all the elements required for a thriving renewable energy business, including plenty of sunshine, strong winds, and, in specific locations, mighty rivers. The area will likely acquire between 22% and 26% of all the solar energy that strikes the globe. This equates to an annual solar energy output potential per square kilometer equal to 1 to 2 million barrels of oil. The potential for solar energy in MENA is regarded as being exponentially greater than the combined potential of all other renewable resources. It is even sufficient to supply the present demand for power worldwide. The MENA region has substantial wind potential in addition to solar potential. The potential for wind energy in Egypt alone is several thousand MW. Wind speeds are among the world’s greatest in Morocco, Egypt, and Tunisia. In conclusion, the MENA countries are attempting to achieve the targets of COP26 and are willing to be advanced enough to counter the environmental challenges. Moreover, Egypt has hosted the COP27, which further motivated the MENA region to put more resources in the fight against environmental damages.

Policymakers are advised to make reforms regarding the legislative framework to attract private sector investment so that the renewable energy sector can achieve its full potential. It is a fact that renewable energy generation is costly. MENA countries must attract foreign investors to invest in this sector. Furthermore, environmental technology should also be promoted and used on a large scale and be easily accessible to the general public. As tourism is one of the major sources of environmental pollutions, however, the policy makers should formulate the policies to minimize the role of tourism in environmental externalities. As such, the governments should promote green tourism by incorporating the energy-efficient means of transport.

## Figures and Tables

**Table 1:** Sources of studied variables and multicollinearity analysis.

**Section A**			
**Variable**	**Code**	**Source**	**Measure**
Ecological footprint: per capita consumptions	EF	Global Footprint Network (GFN)	Global hectares
Urban population	UP	WDI	Measures the annual growth of urban population
GDP per capita (constant 2015 US$)	EG	WDI	The measure of economic growth
Environmental technologies	ET	OECD	Measures countries and firms’ innovative performance and design policies
Renewable energy consumption (% of total final energy consumption)	RE	WDI	Measures the total final consumption of energy
Difference between inbound and outbound tourist	Tourism	WDI	Measures tourism
**Section B**			
Multicollinearity analysis			VIF
UP			8.26
EG			9.03
ET			6.72
RE			4.68
Tourism			7.20

**Table 2:** Descriptive statistics.

	EF	UP	EG	ET	RE	Tourism
Mean	2.765	26.846	4.393	27.749	16.183	3.358
Median	2.804	26.765	4.309	27.696	15.010	3.365
Maximum	3.547	27.999	5.024	29.368	24.982	4.783
Minimum	2.278	26.265	4.215	27.133	9.468	2.251
Std. Dev.	0.254	0.348	0.210	0.372	5.174	0.642
Skewness	0.475	1.172	1.850	2.687	0.483	0.261
Kurtosis	4.525	5.098	5.775	13.167	1.910	2.410
Obs	30	30	30	30	30	30

**Table 3:** Diagnostics of variables.

CDS Tests		
Variables	Pesaran’s CD	Pesaran Scaled—LM
EF	14.862 ***	56.642 ***
UP	42.132 ***	26.911 ***
EG	63.051 ***	62.034 ***
ET	73.294 ***	49.627 ***
RE	62.021 ***	69.421 ***
Tourism	32.575 ***	43.688 ***

Notes: *** represents the level of significance at 1%.

**Table 4:** Test for slop homogeneity.

	Model 1		Model 2		Model 3	
Variables	Δ	Adj Δ	Δ	Δ Adj Δ	Δ	Adj Δ
EF	21.336 ***	25.306 ***	17.965 ***	29.703 ***	27.930 ***	19.522 ***
UP	15.842 ***	29.101 ***	28.140 ***	20.034 ***	34.781 ***	24.783 ***
EG	31.947 ***	33.927 ***	19.542 ***	21.649 ***	24.893 ***	32.235 ***
ET	21.450 ***	29.344 ***	36.309 ***	22.720 ***	36.016 ***	17.291 ***
RE			21.002 ***	29.833 ***	23.604 ***	24.437 ***
Tourism					19.207 ***	22.104 ***

Notes: *** denotes rejection of the null hypothesis of homogeneous slopes at 1%—significant evidence of slope heterogeneity.

**Table 5:** Unit root test.

CADF 1st Diff.			
EF			−3.027 ***
UP			−7.881 ***
EG			−5.065 ***
ET			−4.783 ***
RE			−7.300 ***
Tourism			−6.545 ***
	Model-1	Model-2	Model-3
CIPS (1st difference)	−4.930 ***	−5.721 ***	−5.942 ***

Notes: *** indicates the level of significance at 1%.

**Table 6:** Westerlund co-integration test.

Statistics	Model 1	Model 2	Model 3
Gr	0.001	0.003	0.000
Ga	0.000	0.001	0.000
Pr	0.001	0.010	0.000
Pa	0.020	0.004	0.000

Notes: *p*-values are reported, where <0.01, <0.05 and <0.10 represents the level of significance at 1%, 5% and 10%, respectively.

**Table 7:** Machado, Santos and Silva (2019), Quantiles via Moments.

Quantile	0.1	0.2	0.3	0.4	0.5	0.6	0.7	0.8	0.9	Location	Scale
Model 1											
UP	−0.129 *	−1.741 *	−2.398 *	−1.253 *	0.301 ***	1.573 **	7.684 **	4.663 ***	0.778 ***	6.234 **	−8.621
EG	2.620 *	1.044	2.271 *	0.834 **	0.326 **	5.891 ***	9.344 ***	7.554 **	3.543 ***	9.854 **	3.976
ET	0.824	0.893	−0.384	−0.961 *	−0.184 *	−1.205 ***	−1.564 ***	−0.002 **	−4.665 ***	−5.331 ***	0.880
Constant	13.093 **	21.028 *	23.340 ***	18.164 ***	10.873 ***	8.291 ***	2.098 **	9.763 ***	11.533 **	21.932 ***	7.743
Model 2											
UP	−5.103 **	−3.098 **	3.115 *	6.730 **	1.560 **	2.845 **	1.777 ***	4.539 **	2.221 **	0.800 **	0.392
EG	9.492 *	9.664 *	6.843 **	4.602	7.088 *	3.001	8.562 **	9.005 ***	0.804 ***	6.554 ***	2.841
ET	6.893	0.843	−7.406 **	−8.004 **	−0.230 ***	−5.983 **	−7.560 **	−2.543 ***	−3.900 ***	−0.663 **	−0.783
RE	−3.150	−5.783	−9.101	−1.456	−3.509	−1.117	−4.001 *	−5.444 **	−7.001 **	−4.115 *	7.532
Constant	17.503 ***	25.420 **	16.673 **	13.563 ***	11.908 ***	15.873 ***	6.162 ***	9.778 **	13.472 **	8.143 ***	11.419
Model 3											
UP	−1.674 **	−2.893 **	−7.345 *	−5.320 **	−4.763 **	3.541 **	1.443 **	9.411 **	0.783 ***	1.564 **	2.343
EG	0.655 **	0.562 **	7.450 **	4.332 **	2.549	1.665 **	0.659 *	3.709 **	5.101 **	4.378 ***	3.901
ET	−4.593	−8.203	−9.633	−1.879	−3.709	−4.932 **	−2.789 **	−9.880 ***	−1.663 **	−0.511 **	0.551
RE	−5.294	−7.505	−2.517	−1.004	−9.722	−8.005	−0.556 **	−0.002 *	−6.009 **	−3.981 *	8.200
Tourism	7.503 *	5.723	4.280	3.250	1.300 ***	2.443 **	−6.41	−3.34	1.004 **	7.120 **	0.562
Constant	27.299 **	13.999 **	20.984 **	19.435 **	7.532 ***	11.531 ***	21.333 ***	15.555 ***	18.444 ***	9.404 ***	11.093

Notes: ***, **, * represents the level of significance at 1%, 5% and 10%.

**Table 8:** Driscol Karaay Regression results.

Variables	Model 1	Model 2	Model 3
UP	1.752 *	1.562 ***	1.693 ***
EG	1.343 ***	1.871 ***	2.304 ***
ET	−0.052 *	−0.709 **	−0.671 **
RE		−0.514	−0.306
Tourism			0.418 **
Constant	36.092 ***	28.058 ***	43.436 ***

Notes: ***, **,* represents the level of significance at 1%, 5% and 10%.

**Table 9:** Estimation of models with multiple thresholds.

**Threshold-1**	**Model 1**	**Model 2**	**Model 3**
Threshold value	11.251	11.428	11.465
RSS	13.725	14.375	13.106
MSE	0.432	0.020	0.013
F-statistics	134.378	132.242	139.257
Prob	0.001	0.001	0.002
95% CI			
Lower			10.071
Upper			10.109
**Threshold-2**	**Model 1**	**Model 2**	**Model 3**
Threshold value	12.762	11.428	12.469
RSS	9.287	9.264	9.982
MSE	0.002	0.001	0.004
F-statistics	25.261	27.182	23.561
Prob	0.246	0.266	0.653
95% CI			
Lower			9.734
Upper			10.849
**Threshold-3**	**Model 1**	**Model 2**	**Model 3**
Threshold value	9.043	9.036	9.829
RSS	10.301	10.278	9.248
MSE	0.002	0.001	0.001
F-statistics	18.281	17.324	25.264
Prob	0.289	0.287	0.735
95% CI			
Lower			8.909
Upper			9.267

**Table 10:** Threshold panel regression estimates using threshold-1.

	Threshold Variable		Control Variables			
Model 1	Urban ≤ 10.010	Urban > 10.160	EF	ET	RE	Tourism
	0.542 ***	0.573 ***	1.562 ***	−0.971 *		
Model 2	Urban ≤ 10.045	Urban > 10.295				
	0.394 **	0.576 ***	1.286 **	−0.261 *	−0.082 *	
Model 3	Urban ≤ 10.078	Urban > 10.174				
	0.571 ***	0.547 ***	1.509 ***	−0.262 **	−0.015 *	0.280 **

Notes: ***, **, * represents the level of significance at 1%, 5% and 10%.

**Table 11:** Juodis, Karavias and Sarafidis (2021) [39] Granger non-causality test.

Null Hypothesis	HPJ Wald Test	Coeff
UP to EF	96.267	2.278 ***
EF to UP	120.272	0.245 ***
EG to EF	278.354	1.233 ***
EF to EG	622.347	0.378 ***
ET to EF	106.427	−1.310 ***
EF to ET	147.850	0.393 ***
RE to EF	16.224	−0.027 **
EF to RE	13.348	0.093 **
Tourism to EF	197.675	0.286 ***
EF to Tourism	285.498	0.459 ***

Notes: ***, ** represents the level of significance at 1% and 5%.

**Table 12:** Driscol Karaay Regression results (Post Vision 2030).

Variables	Model 1	Model 2	Model 3
UP	0.016	−0.012 **	−0.038 **
EG	1.182 ***	1.195 ***	1.166 ***
ET	−0.630 ***	−0.972 **	−1.021 **
RE		−0.004 *	−0.002 *
Tourism			0.648 **
Constant	18.762 ***	26.286 ***	32.380 ***

Notes: ***, **, * represents the level of significance at 1%, 5% and 10%.

## Data Availability

All relevant data is included in the paper.
